# Prevention and Control of Catheter-Associated Urinary Tract Infection (CAUTI): A Patient Safety and Quality Improvement Project

**DOI:** 10.7759/cureus.72105

**Published:** 2024-10-22

**Authors:** Rhez Plando, Lina Obaid, Ahmad S Al Baker, Omar Khan, Mariano Solatorio, Bryan J De Leon, Vincent M Tabasin, Ruby A Obsioma

**Affiliations:** 1 Department of Nursing, Sultan Bin Abdulaziz Humanitarian City, Riyadh, SAU; 2 Department of Infection Control, Sultan Bin Abdulaziz Humanitarian City, Riyadh, SAU

**Keywords:** care bundles, catheter-associated urinary infection (cauti), hospital acquired infection, houdini, patient safety, urinary tract infection

## Abstract

Introduction: This patient safety and quality improvement report outlines the successful reduction of catheter-associated urinary tract infections (CAUTI) through a comprehensive interdisciplinary team approach. It emphasizes the implementation of best practices and the involvement of both patients and their families in catheter care. The project was conducted at Sultan Bin Abdulaziz Humanitarian City, the largest rehabilitation facility in the Middle East, with 511 beds and over 20 inpatient units.

Aim: The primary objective of this project was to assess gaps in current CAUTI prevention practices and implement effective strategies to reduce infection rates. By adopting quality improvement methodologies from the Institute of Healthcare Improvement (IHI) and the Deming Four-Stage Cycle, the aim was to prevent CAUTIs and improve patient safety and clinical outcomes.

Method: Urinary catheters, commonly used for patients with neurogenic bladder dysfunction and sacral deep pressure injuries, posed a high risk of infection, with a daily infection risk of 3-7%. Despite available preventive measures, the facility experienced a high average CAUTI rate of 1.28/1000 catheter days from 2021 to mid-2023. This project involved the systematic identification of gaps, the adoption of evidence-based practices, and the engagement of healthcare staff, patients, and families in rigorous CAUTI prevention efforts.

Result: The project resulted in a significant reduction in the CAUTI rate, decreasing from 1.28 infections per 1000 catheter days pre-intervention to 0.42 infections per 1000 catheter days post-intervention. This improvement highlights the effectiveness of collaborative efforts and evidence-based interventions in controlling and preventing CAUTIs.

Conclusion: The findings of this project demonstrate that CAUTI prevention is achievable through a coordinated approach involving healthcare providers, strong evidence-based interventions, and the active participation of patients and families. This collaborative model led to better patient safety outcomes and significantly reduced CAUTI rates, showing the importance of comprehensive care strategies in mitigating preventable patient harm.

## Introduction

Catheter-associated urinary tract infection (CAUTI) is the most common healthcare-associated infection (HAI) and a leading cause of secondary bloodstream infections. Despite many advances in diagnosis, prevention, and treatment, CAUTI remains a significant healthcare burden [1]. CAUTIs occur in patients with an indwelling urinary catheter, defined by the Centers for Disease Control and Prevention (CDC) as a drainage tube inserted into the urinary bladder through the urethra for more than two calendar days (with day 1 being the day of placement). One of the most significant contributing factors to the development of CAUTI is the duration of urinary catheter use. Each day an indwelling urinary catheter remains in place increasing a patient's risk of acquiring CAUTI by 3-7% [2]. Therefore, timely removal of Foley catheters when they are no longer clinically indicated can profoundly impact the prevention and control of CAUTI.

Urinary catheters are widely used at Sultan Bin Abdulaziz Humanitarian City (The City), the largest rehabilitation facility in the Middle East, with a capacity of 511 beds and more than 20 nursing inpatient units. Although CAUTI preventive measures are in place, such as system alerts to identify all patients with urinary catheters, protocols for nurse-driven removal of unnecessary catheters, and CAUTI care bundles, a high incidence persisted from 2021 until the second quarter of 2023.

## Materials and methods

This patient safety and quality improvement project was designed to reduce the incidence of CAUTI by focusing on an interdisciplinary team approach and the use of reliable, recommended evidence-based prevention strategies. All patients with urinary catheters were engaged in the project. Primary interventions included reinforcing registered nurses’ compliance with the HOUDINI process, a protocol that allows them to remove a catheter when it is no longer indicated. The HOUDINI is an acronym where H represents hematuria, gross O represents obstruction, U represents urology, abdominal, gynecological, or perineal surgery, D represents decubitus ulcer, stages 3 and 4 (sacral), I represents input/output measurement, N represents nursing (end of life), and I represents immobility. Additional focus areas through the primary interventions include the CAUTI care bundle, the utilization of a closed drainage urinary system, the use of alternative methods for indwelling catheterization, and involving staff, patients, and families to increase awareness of CAUTI prevention and control. The IHI model for improvement was adapted for this project, followed by testing the changes using Plan-Do-Study-Act (PDSA) cycles. PDSA is a four-step cycle that allows for implementing change, solving problems, and continuously improving processes. Its cyclical nature enables ongoing improvement [3].

Planned interventions: strategy

To mitigate the increasing rate of CAUTI, a patient safety and quality improvement project was initiated in May 2023, involving an interdisciplinary team of physicians, the continence nurse specialist, unit champion/link nurses, clinical resource nurses (CRN), infection prevention and control nurses (IPCN), nursing leadership, and the quality profession. Specific roles and responsibilities were defined to ensure accurate, effective, and efficient execution of the project. The overall approach began by assessing current practices and CAUTI trends and identifying gaps in catheter care guidelines. After thorough brainstorming, gaps were identified, including nursing staff non-adherence to the HOUDINI protocol, poor compliance with the CAUTI care bundle, and a lack of a closed urinary drainage system. Subsequent actions led to the development of a project charter, a formal document outlining the goals and objectives, scope, inclusion and exclusion criteria, milestones, stakeholders, and the quality of methodology applied.

The PDSA tool, a four-stage problem-solving approach [3], was employed by the project team members. Small-scale interventions were initially implemented as a pilot project in each cycle, allowing the team and nursing units to address any concerns or problems that arose during the implementation of the suggested interventions. This approach also provided the team with the opportunity to assess the interventions before distributing them to all nursing units and to refine them before proceeding to the next cycle.

Implementation of PDSA cycles

PDSA Cycle 1

Comprehensive in-service training program: Nursing staff education was delivered by the continence nurse specialist to enhance awareness, understanding, and competency in the prevention and control of CAUTI. The education program included: 1) review and strict adherence to the HOUDINI process; 2) appropriate techniques and procedures for urinary catheter insertion; 3) proper care and management of indwelling catheters; and 4) alternatives to indwelling urinary catheters.

Education for new staff was also conducted, and the role of the unit CAUTI champion/link nurses was strengthened as a direct resource at the bedside for their colleagues regarding catheter care. They worked closely with the continence nurse specialist to oversee and ensure proper implementation of CAUTI prevention strategies.

Introducing the HOUDINI process as best practice: With the primary aim of reducing indwelling urethral catheterization, we adapted an instrument to facilitate the early removal of catheters when no longer clinically indicated. The HOUDINI process, developed by Adams et al. (2012) [4], is a nurse-driven protocol for the timely removal of urinary catheters and a well-documented evidence-based tool that allows registered nurses to review and assess patients’ continued need for indwelling catheter use. When none of the above indications apply to the patient, nurses have the autonomy to remove the catheter without a physician’s order [5]. Registered nurses received comprehensive training on the correct usage of the tool through in-depth education sessions, both virtual and face-to-face. Focused group and unit-specific education programs were also conducted to support and boost their confidence, competence, and decision-making skills regarding when to remove an indwelling catheter. To promote sustainable change, it is essential to make ideas “sticky,” or easy to remember [6]. To facilitate this, a poster of the HOUDINI tool was placed in each nursing unit to aid nurses in comprehending and recalling the information.

**Figure 1:**
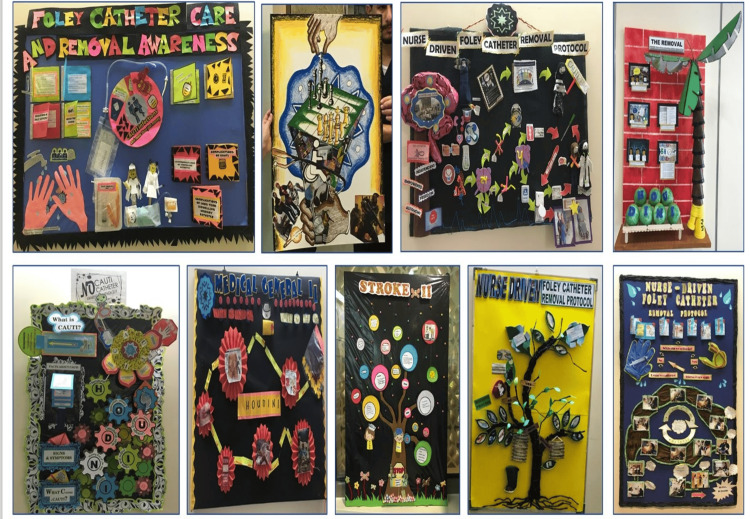
Posters of “No Catheter No CAUTI” campaign CAUTI: catheter-associated urinary tract infection

To further raise awareness and acclimate staff to the HOUDINI process, the project team launched a “No Catheter, No CAUTI” campaign (Figure 1) before its full implementation. Alongside the campaign, a “Nurse-Driven Foley Catheter Removal Protocol Story Board and Decision Tree Making Competition” was conducted to stimulate and enhance registered nurses’ critical thinking and decision-making skills regarding the removal of urethral catheters when they are no longer indicated. Moreover, fostering engagement among staff helps them feel involved and invested in the new changes, rather than overwhelmed by unfamiliar tools and processes. This sense of ownership reduces resistance to change and empowers them to embrace new practices with confidence. Additionally, an education session was held with physicians on the HOUDINI process to enhance the enculturation and foster buy-in.

PDSA Cycle 2

Adherence to the HOUDINI process: Nurses’ compliance with the HOUDINI tool was monitored through documentation reviews on patient medical records to reflect the removal date and time, and moreover, the patient follow-ups by the continence nurse specialist. The use of HOUDINI was further integrated into our continence care and management pathway and related policies and procedures to support nurses in their decisions regarding the insertion and removal of urethral catheters.

Strict compliance with the CAUTI care bundle: Care “bundles” are simple sets of evidence-based practices that, when implemented collectively, improve the reliability of their delivery and enhance patient outcomes [7]. The “Care Bundle” approach is a strategy wherein a series of actions are performed in a prescribed manner to decrease the incidence of CAUTI [8]. Through the efforts and guidance of the IPCNs, strict implementation of the catheter care bundle was reinforced among all nursing staff, and compliance was rigorously monitored through consistent surveillance. Care bundle compliance was monitored through daily unit rounds, especially in high-risk areas such as the wound care unit and intensive care unit. Validation of care bundles is performed weekly by the IPCNs through direct observation of patients with indwelling urethral catheters, with real-time feedback provided to the staff as part of the quality improvement initiative.

**Figure 2:**
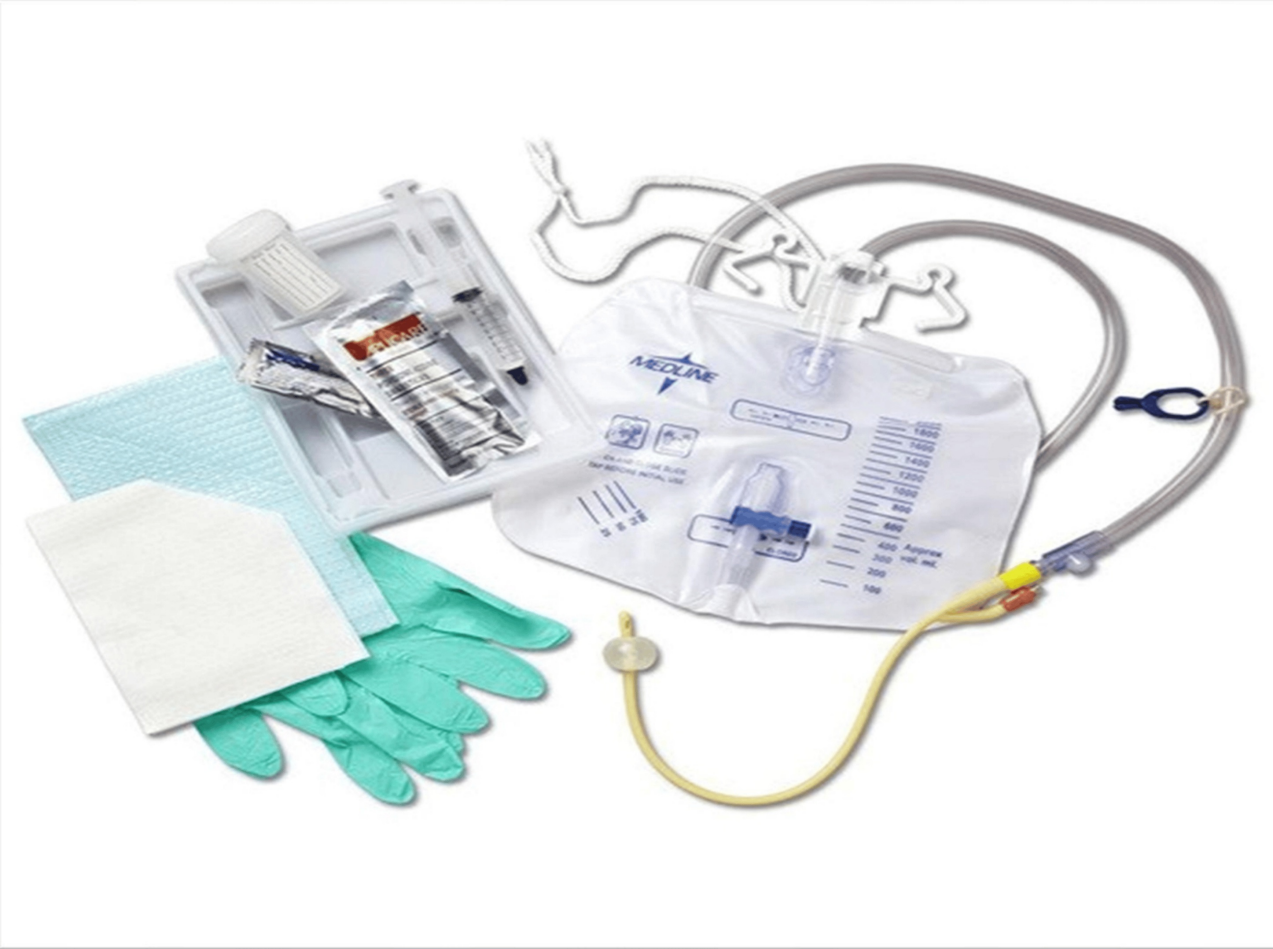
Closed drainage indwelling catheter system

Lack of a closed urinary drainage system: A closed catheter drainage system is an aseptic system in which the path from the tip of the catheter inserted into the bladder to the bag that collects urine is closed and should not be disconnected. This structure is designed to eliminate the inoculation of the urinary tract with bacteria via the catheter drainage tubing and collection bag. The introduction of the closed drainage indwelling catheter system (Figure 2) was a significant advance in the prevention of urinary catheter-related infections [9]. In a quasi-experimental five-month study, the authors concluded that the utilization of a closed drainage system with an anti-backup flow valve significantly reduces the rate of urinary tract infections [10]. This reduction has been attributed to the elimination of the risk of “human factors” during catheter insertion, care, and maintenance. The project team fervently proposed to the Director of Nursing and the Supply Chain Management Director to make the closed urinary drainage system available at our facility. With their support and commitment to excellent patient care and safety, the proposal was approved, and the urinary system is now in use.

PDSA Cycle 3

Alternative use of indwelling urinary catheters: The 2009 CDC Guidelines for the Prevention of CAUTI recommend that “clinicians should consider external catheters as an alternative to indwelling urinary catheters in cooperative male patients without urinary retention or bladder outlet obstruction” [2]. In adherence to this, the team collaborated with physicians to advocate for the use of external catheters instead of indwelling catheter placement. Additionally, external catheters are considered more comfortable and allow for greater mobility compared to indwelling catheters. After careful discussion and agreement, the team provided education to nursing staff to ensure proper and appropriate implementation of the new practice.

Regular internal audit programs: A joint effort was orchestrated by nursing leadership and the CRN through random unit visits to assess nursing staff compliance with CAUTI care guidelines, moreover, to hear their challenges and needs pertaining to the new changes in nursing practices and to tackle issues immediately as needed. Performance feedback was provided to the managers and nursing staff, followed by on-the-spot education and reinforcement of appropriate catheter care.

Patients and their families were also included during these visits. They were educated on the necessity and rationale for catheterization and provided with information on CAUTI prevention techniques. Educational materials with a QR code were made available in each patient's room for easy access. To further strengthen their engagement, an evidence-based tool, a urinary catheter passport, was provided to patients being discharged with indwelling urinary catheters. The passport serves as patient-held written documentation of education and care plans on catheter care and management, empowering both patients and healthcare providers. This tool bridges the existing information gap, improves care, promotes self-care, and helps patients adjust to living with their catheter, especially when complemented by ongoing input from a nurse or other health professional. More significantly, the passport can greatly reduce the risk of CAUTI and prevent costly readmissions and unnecessary antibiotic use [11]. The passport was designed and produced in both Arabic and English to ensure effective communication and understanding among the diverse population served.

Analysis

Two parameters were used to analyze the progress of the project interventions before and after implementation.

First, the incident rates of CAUTI were evaluated using statistical process control process behavioral charts [12] to assess the outcomes of the project interventions. The team applied established rules to differentiate between common cause variation and special cause variation as follows: Rule One: any data point outside the limit; Rule Two: eight consecutive points on the same side of the central line; Rule Three: three out of four consecutive data points that are closer to the same limit than they are to the central line.

If any of the three rules occurred, it was considered a special cause variation and denoted with red dots in the displayed process behavioral chart, which guided and supported the team to react less, lead better, and improve more.

The project was deemed successful due to two special cause variations in the process behavioral chart indicating improvement, with a p-value of less than 0.05.

Second, statistical analysis of the p-value between pre-intervention and post-intervention data, along with descriptive analysis of the central tendency measures, was conducted.

## Results

Result: project outcomes

**Figure 3:**
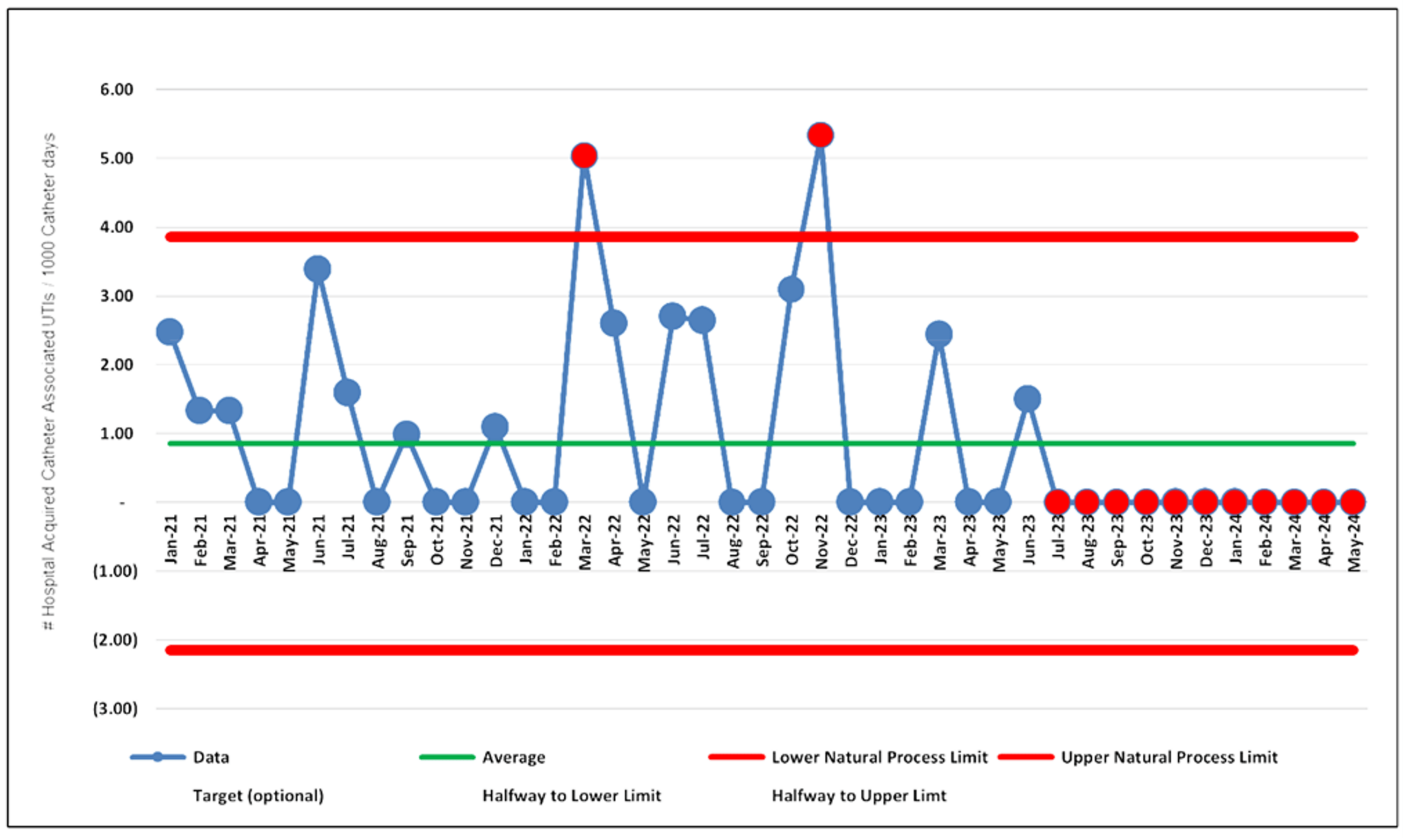
The hospital-acquired catheter-associated UTIs per 1000 catheter days process behavioral charts UTIs: urinary tract infections

The team examined the results of PDSA cycles 1-3 for quantifiable outcomes across the nursing units. The graph below (Figure 3) displays significant improvement from July 2023 to May 2024, characterized by eight consecutive points on the same side of the central line (refer to Rule Three in the Analysis section) in the hospital-acquired catheter-associated UTIs per 1000 catheter days process behavioral charts.

The descriptive statistical analysis data supported the project outcomes. The table below shows a statistically significant difference in the CAUTI rate between pre-interventions and post-interventions, calculated with a p-value of 0.04 (Table 1), with a confidence interval of 95% (Table 2).

**Table 1:** P-values in pre-interventions and post-interventions

t-test: paired two samples for means	Pre-interventions	Post-interventions
Mean	1.284772727	0.421363636
Variance	2.166418184	1.557450433
Observations	22	22
P (T≤t) two-tail	0.044847731	

**Table 2:** Central tendency measures

Central tendency measures	Pre-interventions	Post-interventions
Mean	1.284772727	0.421363636
Standard error	0.313804961	0.266069918
Median	1.042	0
Mode	0	0
Standard deviation	1.471875737	1.247978539
Sample variance	2.166418184	1.557450433
Sum	28.265	9.27
Count	22	22
Confidence level (95.0%)	0.652593142	0.553322686

## Discussion

As a rehabilitation facility where most patients develop neurogenic bladder dysfunction due to traumatic or non-traumatic central neurologic disorders, and with one nursing unit dedicated solely to wound care patients with deep pressure injuries on sacral and gluteal areas, the use of indwelling urethral catheters is often unavoidable. The recurring and alarmingly high CAUTI rates from 2021 until the second quarter of 2023 warranted a thorough investigation, as this directly affects the delivery of quality and safe patient care. The team believes that one of the most effective measures to alleviate the problem is through a patient safety and quality improvement project, which refers to activities that use data-based methods - some developed in manufacturing industries - to bring about immediate improvements in healthcare delivery. Quality improvement methods enable healthcare personnel to implement changes systematically, measure and assess the effects of those changes, and adjust until they are satisfied with the results [13]. With this initiative, carried out over six months from June 2023 until December 2023, we found that the collaborative engagement of all healthcare personnel and the implementation of multiple strategies resulted in a significant reduction in catheter utilization days and CAUTI rates. Physicians, ICNs, nursing leaders and educators, registered nurses, patients, and their families all played significant roles in preventing CAUTIs. Similarly, applying multiple interventions based on credible recommendations successfully achieved positive outcomes in CAUTI prevention.

The significant reduction in CAUTI rates can be directly attributed to the use of the HOUDINI process, which restricts catheter placement and limits catheter utilization days. Patients admitted to our facility with indwelling urethral catheters are assessed using this tool, and after 24 hours of continued assessment, nurses are empowered to independently remove the catheter if the patient does not meet the HOUDINI criteria. Providing bedside nurses with an evidence-based protocol driven by specific patient indications and diagnoses allows them to practice autonomously and promptly remove indwelling urinary catheters, which may result in decreased device days and a reduced incidence of CAUTIs [14]. Moreover, an interventional study of a hospital-wide, multidisciplinary program to reduce urinary catheter use and CAUTIs on all patient care units in a 300-bed teaching hospital in Connecticut resulted in a 50% reduction in catheter use and a 70% reduction in CAUTIs over a 36-month period. The study concluded that aggressive implementation of the nurse-directed catheter removal protocol was associated with lower catheter use rates and reduced infection rates [15].

Compliance with the CAUTI care bundle has also impacted the outcome of this project. It operates on an all-or-nothing compliance score, measured as either 100% or 0%. To achieve 100%, all evidence-based components of the bundle must be implemented. If one component is not in place, a score of zero is assigned to the unit. One of the studies emphasized that even though a few elements of the care bundle were already in practice, adherence to all elements as a bundle brought a significant decrease in CAUTI. Adherence to each element will have some influence in reducing CAUTI in terms of reducing the device utilization ratio and average catheter days per patient. Thus, implementing care bundles and auditing adherence to each element should be included as a part of routine hospital infection control committee practices [16]. The Institute for Healthcare Improvement, USA, developed the concept of bundles to help deliver bedside care more reliably and effectively. A care bundle is a powerful tool for improving patient care and outcomes [17].

The utilization of a closed-drainage urinary system, in alignment with CDC recommendations, has significantly contributed to the reduction of CAUTI rates following the project's implementation, likely due to a streamlined process for aseptic catheter insertion. Since the closed system catheter is a self-contained, pre-lubricated catheter housed within its collection bag, the entry of pathogenic microorganisms is hindered during catheter insertion, care, and maintenance. In a prospective study of 676 patients with indwelling urethral catheters, the incidence of bacteriuria in those managed with continuously closed urinary drainage was 23%, significantly lower than the historic incidence of 95% for open urinary drainage. Based on this study, the use of closed catheter urinary drainage systems became the standard of care [18].

The team was also successful in advocating for alternative bladder management methods to avoid catheter placement, which significantly reduced urinary catheter utilization. Currently, our urinary management protocol stipulates that indwelling catheters are inserted only when medically indicated; otherwise, patients remain catheter-free. A prospective, randomized, unblinded, controlled trial concluded that the use of external catheters is less likely to lead to bacteriuria, symptomatic UTIs, or death compared to indwelling catheters. Additionally, external catheters [19] are widely used in chronic care facilities because they avoid urethral trauma and improve patient comfort and mobility compared to indwelling catheters [20].

Another highlight of the project was the initiation of regular internal audit programs conducted by nurse leaders, who surveyed patients with indwelling urethral catheters and assessed nursing staff adherence to catheter care guidelines. This also provided nursing leaders with the opportunity to educate patients and families on catheter care. Audits and feedback - providing healthcare professionals with timely data about their performance - have proven effective in improving the quality of care [21]. The engagement strategies that have been used to engage nursing staff and unit leaders played a crucial role in facilitating the smooth implementation of interventions, significantly reducing resistance to change among staff. Moreover, these strategies fostered a culture of open communication, enabling consistent collection of staff feedback to address their needs and challenges effectively [22]. Additionally, actively listening to the voices of patients and their families and educating them about new practice changes were vital in supporting the successful implementation of the HOUDINI process, ultimately leading to improved patient care and outcomes [23].

Last, the provision of an evidence-based urinary catheter passport has greatly enhanced patients’ knowledge and understanding of self-care, especially after being discharged with indwelling urinary catheters. The catheter passport can also bridge the existing information gap, improve care, and help patients adjust to their catheter, especially if complemented by ongoing input from a nurse or other health professional [11]. One patient remarked, “Now the thing is more easy after I received information from the passport. This makes me feel comfortable and confident in managing my catheter at home. I will also share this with my family members so they will also learn how to take care of my catheter.” Patients involved in the decision-making process are more likely to comply with their treatment and management of care, leading to more positive outcomes [24].

Following this patient safety and quality improvement project, the City now has more tangible and comprehensive measures to prevent CAUTI, as evidenced by consistent zero CAUTI cases and a sustained rate post-implementation of the initiative. This is a strong testament to our commitment to delivering high-quality and safe patient care. Considering it is widely known that most projects fail due to the absence of a comprehensive sustainability plan, a comprehensive plan (Table 3) was developed by the core team of the project [25].

**Table 3:** Sustainability plan CAUTI: catheter-associated urinary tract infection

The actions of the plan included the following:	
1	Automating the HOUDINI tool (Supplemental material 1) through the use of the Health Information System.
2	Strengthening the involvement of patients and their families through the use of the CAUTI Passport.
3	Enhancing in-service education materials through the use of international e-learning platforms.
4	Revising hospital policies and procedures based on best practices and evidence.
5	Integrating catheter care guidelines into the Continence Care and Management Pathway.
6	Reviewing CAUTI care bundle compliance rates on a monthly basis.
7	Conducting regular internal audits by nursing leadership and CRN to assess nursing staff compliance with CAUTI care guidelines.

Limitation and lessons

The project was conducted entirely in a rehabilitation facility in the Middle East, which limits the generalizability of the findings. Additionally, these outcomes cannot be attributed to a single intervention, as multiple quality improvement strategies were implemented simultaneously.

Invaluable lessons were learned throughout the journey of this project. The involvement of the interdisciplinary team, the support of nursing leadership, the active engagement and commitment of nursing staff, and the application of best practice recommendations all contributed to the success of achieving our goal. It is also important to note that early intervention promotes positive clinical outcomes and enhances patient safety.

## Conclusions

Preventing and controlling CAUTI is an ongoing challenge for patient safety. However, the collective efforts of all healthcare professionals responsible for providing high-quality patient care, along with the robust implementation of reliable prevention strategies and the empowerment of staff, patients, and families, can substantially impact positive clinical outcomes and reduce the risk of urinary tract infections.

## Figures and Tables

**Figure 4:**
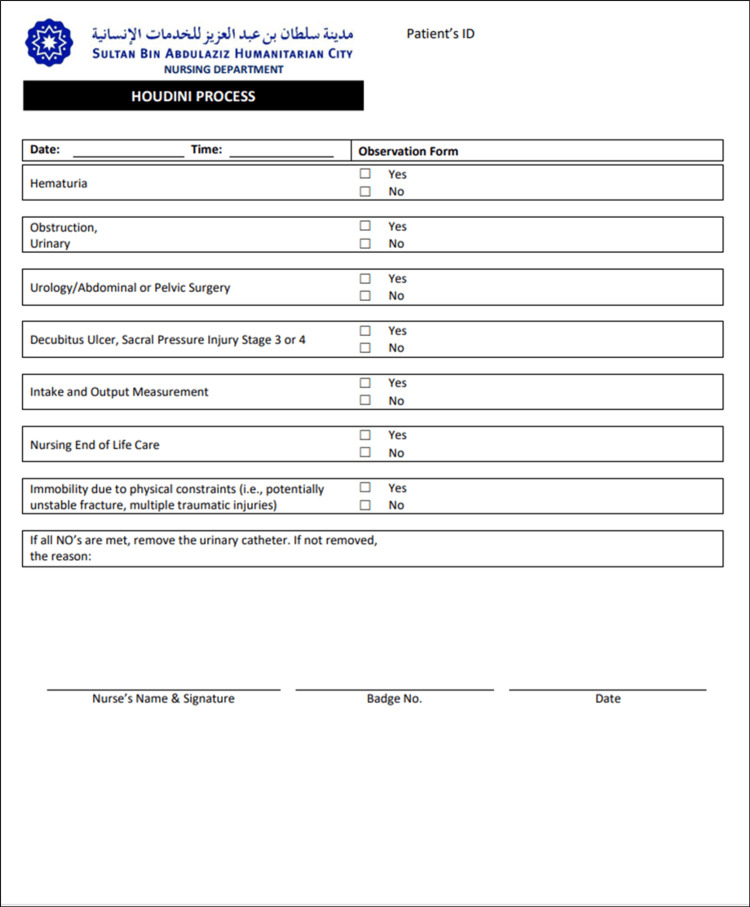
HOUDINI tool through the use of health information system Reference: [4]

## Appendices

Supplemental material 1 
